# Use of extracorporeal membrane oxygenation during resection of recurrent laryngeal papillomatosis: A case report

**DOI:** 10.1097/MD.0000000000033949

**Published:** 2023-06-02

**Authors:** Wenyang Wang, Zhirui Zhu, Wenlong He, Yaoqin Hu, Qiao Zeng

**Affiliations:** a Department of Anesthesiology, The Children’s Hospital, Zhejiang University School of Medicine, Hangzhou, China; b Department of Extracorporeal Circulation and Extracorporeal Life Support, The Children’s Hospital, Zhejiang University School of Medicine, Hangzhou, China.

**Keywords:** airway management, case report, extracorporeal membrane oxygenation, laryngeal papilloma, temperature-controlled ablation radiofrequency

## Abstract

Recurrent laryngeal papillomatosis (RLP) is a common benign tumor in the larynx of children, which is characterized by high recurrence rate and rapid growth, leading to clinical symptoms such as hoarseness and difficulty breathing. Low-temperature plasma radiofrequency ablation (RFA) is the main treatment, but ventilation problems are often encountered during surgery.

**Patient concerns:**

We report a case of a 2-year-old child with RLP who underwent low-temperature plasma RFA with the assistance of a laryngoscope. However, the surgery had to be temporarily stopped due to ventilation difficulties and difficulty in maintaining blood oxygen saturation during the procedure.

**Diagnosis:**

The child was diagnosed with RLP.

**Interventions:**

The child underwent low-temperature plasma RFA supported by laryngoscopy assisted by ECMO.

**Outcomes:**

Despite ventilation problems during surgery, the use of ECMO support helped maintain good oxygen saturation in the child and provided a clear surgical field, enabling the tumor to be quickly and cleanly removed. Therefore, the use of ECMO provided critical support during the surgery.

**Lessons:**

This case highlights the importance of airway management during laryngeal papillomatosis surgery. A thorough airway assessment should be performed before anesthesia, and early use of ECMO can reduce harm to the child and ensure the child’s safety.

## 1. Introduction

Recurrent laryngeal papillomatosis (RLP) is a common benign tumor that occurs in the larynx, primarily in children and adults under 10 years old. This condition is mainly associated with human papillomavirus (HPV) infection. The incidence rate is reported to be 0.18/100,000 in adults and 1.34/100,000 in infants.^[1]^ RLP in children is characterized by multiple lesions, rapid growth, frequent recurrence, and potential spread to the lower respiratory tract. The growth of the tumor may lead to airway obstruction, which can manifest as cough, hoarseness, wheezing, dyspnea, and even life-threatening conditions.^[2]^ Surgical resection is currently the most effective treatment for RLP.^[3]^ Airway management during surgery is often determined by the clinical symptoms and degree of laryngeal obstruction. In this article, we present a case of a child with laryngeal papilloma who underwent low-temperature plasma radiofrequency ablation with a support laryngoscope under extracorporeal membrane oxygenation (ECMO). We describe the detailed process of this procedure.

## 2. Presentation

The patient is a 2-year-old female weighing 12.4 kg who presented to the hospital with a history of laryngeal papilloma for >1 year, and hoarseness and shortness of breath for >1 week. One year ago, the child underwent laryngoscopic laryngeal papilloma resection under general anesthesia and had improvement in hoarseness and shortness of breath after surgery. However, the symptoms recurred and the child underwent surgical resection once per month on average, with the last operation being >1 month ago.

On admission, the child had hoarseness, shortness of breath, and poor breath, with a nasopharyngoscopy revealing a papillary new organism obstructing the airway on the glottic. Physical examination showed a body temperature of 37.3 degrees Celsius, respiratory rate of 30 breaths per minute, pulse oxygen saturation (SpO_2_) of 100% with oxygen inhalation via nasal cannula at 2 L/min, clear breath sounds, visible presence of 3 inspiratory notches, normal cardiovascular examination, and no abnormalities in the rest.

**Figure 1.**
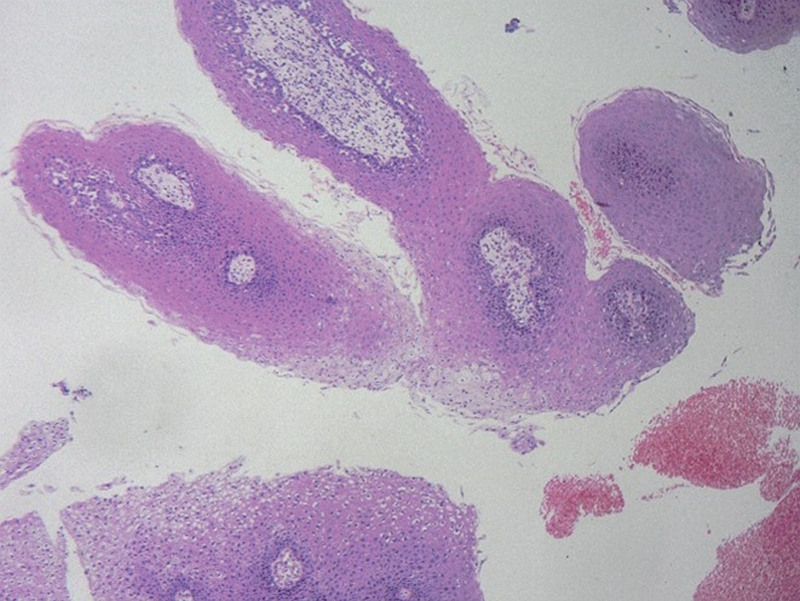
Postoperative pathology.

**Figure 2.**
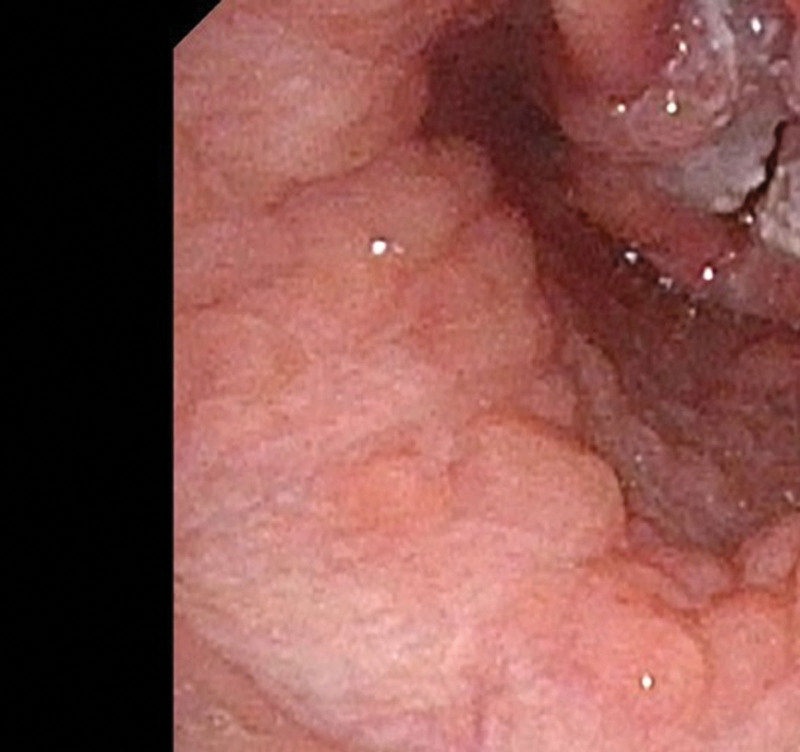
Laryngoscopy.

Laryngoscopy revealed that papillary neoorganisms were obstructing the airway on the glottis, accumulating bilateral ventricular belts, laryngeal ventricles, vocal cord surfaces, and free margins, with only small cracks visible when exhaling. Emergency surgical treatment was therefore chosen. (Figs. 1 and 2 show the pathological suggestion and laryngoscopy findings, respectively).

On the second day of admission, the patient was prepared for surgery to remove the papillomatous tumor in the larynx. Before anesthesia, the patient’s heart rate and blood pressure were stable. the patient exhibited obvious symptoms of respiratory distress, including hoarseness and agitation. The patient’s airway obstruction was graded as level 3, but the patient was able to breathe using a face mask. It was uncertain whether the patient had a difficult airway.

At 15:55, the patient was administered medications including 1 mg of midazolam, 25 mg of propofol, 10 mg of lidocaine, 3 mg of nalbuphine, 0.15 mg of atropine, 10 mg of methylprednisolone, and 2 mg of ondansetron. The laryngoscope was inserted for observation, and intubation was successful without difficulty. Propofol was intermittently administered to maintain the depth of anesthesia, and the surgery was carried out with the patient breathing spontaneously.

At 16:40, the patient’s SpO_2_ rapidly dropped to 85%, and it was difficult to ventilate with a face mask. Laryngospasm was suspected, and 25 mg of propofol was immediately administered. A 3.0# tracheal tube was inserted, and 6 mg of rocuronium was administered as a muscle relaxant. The surgery was continued with intermittent ventilation. However, after the second insertion and removal of the tracheal tube, difficulty was encountered in reinserting the tube. The instrument was withdrawn, the index and middle fingers were inserted into the oral cavity to expand the pharynx, and the tracheal tube was pushed through the glottis.

With the consent of the family, the surgery was temporarily stopped, and the patient was sent to the intensive care unit and used ECMO assistance for the next surgery after a multidisciplinary team consultation.

On day 5, the child was brought to the operating room with an anesthesia induction tube and arterial blood pressure monitoring. The surgery utilized an improved venous artery ECMO auxiliary mode, which included the use of an extracorporeal circulation membrane lung and tubing, an ECMO host, and a centrifugal pump. This system not only met the short-term auxiliary needs during surgery but also greatly reduced the financial burden on the child’s parents. The improved ECMO system utilized several components, including ECMO machines from Maquet in Sweden, a SORIN Heater-Cooler System, a monitoring display from Medtronic in the USA, a membranous lung from Medtronic BBP241 in the USA, a centrifugal drive pump from Maquet in Sweden, and a tubing package for extracorporeal circulation without heparin coating. After the conventional ECMO system was exhausted, 10 g of albumin, 10 mL of sodium bicarbonate, 10 mL of calcium gluconate, 10 mg of heparin, and 1 unit of red blood cells were added to precharge the system, which was then preheated at 37°C.

After general anesthesia was administered, the child was placed in the supine position, with their head turned to the left. A transverse incision was made in the right neck to separate the common carotid artery and the internal jugular vein. The child was given 100 U/kg heparin and a 14 F arterial cannulation (from Medtronic in the USA) was fixed. The speed was set to 2650 rpm, with a blood flow of 1.0 L/min and an airflow of 1.0 L/min. The operation was smooth, with stable vital signs including a heart rate of 110 beats/min, blood pressure of 110/60 (75) mm Hg, SpO_2_ of 100%, and mixed venous oxygen saturation of 82%. Table 1 provides arterial blood gas analysis, which showed no significant abnormalities. The tracheal intubation was removed, and a support laryngoscopic laryngeal papilloma resection was performed. After tumor resection, a 4.0 capsulated pressurized tracheal tube was reinserted, while the ECMO flow was gradually reduced to about 10%. The child’s vital signs remained stable. The ECMO system arteriovenous was clamped, and the arteriovenous cannula and vascular anastomosis repair were removed. After hemostasis, the neck wound was sutured, and the evacuation was smooth. The ECMO ran for a total of 55 minutes, with the operation lasting 40 minutes and the anesthesia time being 205 minutes.

**Table 1:** Blood gas analysis results.

	Before establishing ECMO	After establishing ECMO	After surgery
SvO_2_ (%)	–	70	–
SaO_2_ (%)	99.4	98.7	98.9
SpO_2_ (%)	99	92	99
pH	7.453	7.413	7.487
Hb (g/L)	83	90	103
K^+^ (mmol/L)	3.2	3.9	3.7
PaO_2_ (mm Hg)	138	110	174
PaCO_2_ (mm Hg)	31.6	35.7	31.2
HCO_3−_ (mmol/L)	21.8	22.3	23.4
BE (mmol/L)	−1.6	−1.6	0.3
Lac (mmol/L)	0.8	1.3	1.4

BE = base excess, ECMO = extracorporeal membrane oxygenation, Hb = hemoglobin, HCO_3−_ = bicarbonate ion, K^+^ = potassium ion, Lac = lactic acid, PaCO_2_ = partial pressure of carbon dioxide in artery, PaO_2_ = partial pressure of oxygen in artery, pH = potential of hydrogen, SaO_2_ = arterial oxygen saturation, SpO_2_ = pulse oxygen saturation, SvO_2_ = oxygen saturation in ECMO drainage cannula.

On the second day after the second operation, the child was able to recover spontaneous breathing and the endotracheal tube was removed. The child was discharged on the 4th day with no complications such as bleeding, infection, pneumothorax, or dyspnea after surgery. At discharge, the child’s hoarseness had significantly improved compared to before, and there were no signs of cyanosis or other discomfort. The patient’s family was very satisfied with the consultation process. The child’s parents were very satisfied with the treatment process. But the child had to undergo surgical treatment until the tumor was fully treated and did not recur.

## 3. Discussion

Laryngeal papilloma is the most common noninvasive benign tumor of the larynx in children, which often recurs and has a typical onset age of <4 years old. HPV infection is the primary cause of laryngeal papilloma.^[4]^ The development and prognosis of the disease are closely associated with the level of serum anti-HPV antibody. The disease affects the larynx and trachea, with the primary symptoms being hoarseness and dyspnea, particularly in severe cases with airway obstruction.^[5]^ Given the high risk of surgery and anesthesia, children should undergo fiberoptic laryngoscopy to assess the growth range of the tumor, the degree of tracheal stenosis, scar adhesion, and any tumor blocking the distal end of the tracheotomy tube. If the degree of laryngeal obstruction is 2 degrees or more, surgery should be performed as soon as possible to remove airway obstruction.

The ideal anesthesia for surgery with a support laryngoscope requires stability, safety, fixed vocal cords, good exposure to the surgical field, sufficient space for the operator to operate the instrument, and rapid recovery of various reflexes after the operation. Common airway management methods include intermittent ventilation through endotracheal intubation, high-frequency ventilation, and reserved spontaneous respiration.

Intermittent ventilation through tracheal intubation involves the use of muscle relaxants, deep anesthesia, and a thinner tracheal catheter for ventilation. Disadvantages include potential damage to the glottis due to narrow spaces in which the surgeon operates and short operating times, as well as laryngeal edema that can occur after multiple tracheal intubations. High-frequency ventilation utilizes high-pressure jet air sources to generate airflow and involve more air into the lung tissue, increasing the ventilation volume. It has the advantage of providing sufficient oxygen supply and clear vision but requires skilled anesthesiologists and carries the risk of convulsions, choking, and lung injury. Reserved spontaneous respiration is suitable for children with mild laryngeal obstruction or no obstruction while under deep anesthesia, allowing for quick removal of the subglottic tumor by surgeons. However, it also carries the risk of spasms, suffocation, and carbon dioxide retention.^[6]^

If the operation interval is long, and the degree of laryngeal obstruction is severe, retaining the autonomous breathing mode or intermittent ventilation mode is unsafe. In this case, surgical treatment under ECMO is recommended. ECMO is an effective way to maintain oxygenation in situations where it is not safe or feasible to perform endotracheal intubation or tracheotomy due to severe difficult airway. ECMO provides a clean and clear operation field for surgeons, ensures the stability of hemodynamics and oxygen supply for patients, and enables safe surgical procedures. However, ECMO technology requires specialized equipment and professional teams and carries a high medical cost, along with the risk of complications such as bleeding, thrombosis, infection, limb ischemia, and necrosis at the intubation side.

However, there are some limitations in this study. Although children with RLP who have difficulty in intubation have successfully undergone surgery under ECMO treatment, which has rarely been reported, laryngeal papilloma is prone to recurrence after surgery and cannot be completely cured in a short time. Multiple surgeries are necessary. Although there were no apparent complications with ECMO in this case, the incidence of complications with ECMO is high and could be very dangerous. ECMO is also costly and can impose an economic and psychological burden on patients’ families, making it unsuitable for multiple uses.

## 4. Conclusion

Based on the above case experience, ECMO is a feasible and safe method to assist in the successful completion of critical airway surgery. It is essential to decide whether to use ECMO before anesthesia induction. With further experience and research, more complex surgical procedures can be performed to manage critical airway problems in the future.

## Author contributions

**Conceptualization:** Wenyang Wang, Yaoqin Hu.

Data curation: Qiao Zeng.

Methodology: Wenlong He.

Writing – original draft: Wenyang Wang.Writing – review & editing: Qiao Zeng, Zhirui Zhu, Yaoqin Hu.
